# ‘Drugs That Make You Feel Bad’? Remorse-Based Mitigation and Neurointerventions

**DOI:** 10.1007/s11572-015-9383-0

**Published:** 2015-10-05

**Authors:** Jonathan Pugh, Hannah Maslen

**Affiliations:** 1 Oxford Uehiro Centre for Practical Ethics, University of Oxford, Suite 8, Littlegate House, St Ebbes Street, Oxford, OX1 1PT UK; 2 Oxford Martin School, University of Oxford, 34 Broad Street, Oxford, OX1 3BD UK

**Keywords:** Remorse, Mitigation, Neurointerventions, Memory, Empathy, Authenticity

## Abstract

In many jurisdictions, an offender’s remorse is considered to be a relevant factor to take into account in mitigation at sentencing. The growing philosophical interest in the use of neurointerventions in criminal justice raises an important question about such remorse-based mitigation: to what extent should technologically facilitated remorse be honoured such that it is permitted the same penal significance as standard instances of remorse? To motivate this question, we begin by sketching a tripartite account of remorse that distinguishes cognitive, affective and motivational elements of remorse. We then describe a number of neurointerventions that might plausibly be used to enhance abilities that are relevant to these different elements of remorse. Having described what we term the ‘moral value’ view of the justification of remorse-based mitigation (according to which remorse-based mitigation is justified insofar as mitigation serves as a deserved form of response to the moral value of the offender’s remorse), we then consider whether using neurointerventions to facilitate remorse would undermine its moral value, and thus make it inappropriate to honour such remorse in the criminal justice system. We respond to this question by claiming that the form of moral understanding that is incorporated into a genuinely remorseful response grounds remorse’s moral value. In view of this claim, we conclude by arguing that neurointerventions need not undermine remorse’s moral value on this approach, and that the remorse that such interventions might facilitate could also be authentic to the recipient of the neurointerventions that we discuss.

Consider the following real-life case of Maciej Zientarski^1^:

Zientarski was driving at speeds of over 140 kph and crashed; his passenger was killed, and Zientarski was put into a coma. When he awoke, it became apparent that Zientarski was unable to remember any details about the crash. He was eventually found fit to stand trial, and was found guilty of causing death by dangerous driving. However, Zientarski claimed that he found it impossible to feel any remorse for his crime, since his amnesia precluded him from remembering the events of the crash.

In many jurisdictions, an offender’s remorse is considered to be a relevant factor to take into account in mitigation at sentencing. For example, the 2011 Crown Court Sentencing Survey for England and Wales revealed that remorse was the most commonly invoked mitigating factor for six out of eight offence types.^2^ In such jurisdictions, offenders who are deemed to be remorseful often receive sentences that are less severe than those who do not express remorse. Indeed, remorse can often tip the balance in favour of a non-custodial sentence,^3^ and even make the difference between life and death in capital cases.^4^

Zientarski’s case raises a number of theoretical questions about this practice. Suppose that it had been possible to reverse Zientarski’s amnesia with a neurointervention such as deep brain stimulation. Suppose that Zientarski had volunteered for such an intervention and had come to fully remember the events of the crash. Suppose further that he consequently came to experience remorse for his crime. Should the remorse that Zientarski feels following the intervention in this hypothetical permutation of the case be understood as genuine, and as providing sufficient grounds for mitigation in the same way as naturally-occurring remorse might?

This sort of scenario is perhaps not as fantastic as it might seem. Philosophers have begun to seriously consider ways in which neurointerventions could be used in the criminal justice system. Indeed, neurointerventions are increasingly being used for crime prevention; to take one example, sex offenders in some jurisdictions are required to undergo chemical castration in order to prevent recidivism amongst this group of offenders.^5^ Furthermore, in the US, offenders can be made to take anti-psychotic drugs in order to restore mental faculties that render them competent to stand trial, or to receive punishment.^6^

In this paper, we shall argue that, in some limited cases, neurointerventions could be used to facilitate genuine remorse of the sort that could provide justifiable grounds for mitigating criminal sentences. At the outset, it is important to acknowledge the scope of our argument. First, we shall not here defend the practice of remorse-based mitigation *per se*; rather, we assume that this practice is justified in order to consider whether we should treat an experience of remorse that has been facilitated by a neurointervention differently from naturally-occurring remorse. Second, we do not argue that the criminal justice system ought to use such interventions to facilitate remorse, nor do we defend the practice from all of the moral objections that might be raised against using these technologies in different sorts of scenarios. Rather, in this paper, we consider whether it is philosophically plausible to believe that neurointerventions could be used to facilitate genuine remorse of the sort that might be understood to provide grounds for mitigation *in at least some cases*.

To begin this investigation, we shall, in Sect. 1, sketch a tripartite account of remorse, before going on, in Sect. 2, to provide an overview of neurointerventions that could plausibly have an effect on capacities related to elements of remorse. In Sect. 3, we shall explain a widespread view of the justification of remorse-based mitigation, according to which mitigation is a deserved form of response to the moral value of the offender’s remorse. We shall then go on to consider whether the use of neurointerventions to bring about remorse would undermine this moral value in a way that would make it inappropriate to mitigate the sentence of an offender whose remorse had been facilitated by a neurointervention.

## The Elements of Remorse

Remorse is often simply referred to as an emotion, but emotional experiences can be complex, involving beliefs and desires in addition to feelings and sensations.^7^ Whilst a number of accounts of remorse have been offered in the literature, in this section, we shall provide a sketch of what we take to be a minimal account of remorse, and draw a distinction between cognitive, affective and motivational elements of remorse.^8^ In the interests of brevity, we shall henceforth refer to an agent who has committed a criminal act/omission as ‘the offender,’ and the criminal act/omission that she has committed as ‘X.’

The distinction that we draw between these elements echoes other prominent accounts of remorse. However, whilst we share the view that it is useful to distinguish the different elements of remorse in this manner, we shall suggest below that this approach is limited in one crucial respect. Briefly, the problem is that, in order for remorse to be genuine and to bear moral value, it seems that the elements that we identify below must be related in certain ways, and isolating these elements in the manner of our below discussion can serve to obscure this. We shall develop this thought in Sect. 3.

### The Cognitive Element

Remorse involves certain beliefs, which can be articulated at a sufficiently abstract level to constitute necessary features. If ‘the offender’ is remorseful, she will have both non-evaluative and evaluative beliefs about the event in relation to which she experiences remorse. The non-evaluative beliefs will take the form:

(i) ‘I performed X,’ and
(ii) ‘X affected another identifiable agent.’

In holding these beliefs, the agent accepts causal responsibility for her act, and that this act affected another identifiable agent. This is necessary for remorse: one cannot feel remorseful over the acts of other agents, nor for acts that did not affect others. In remorse, the agent is responding not just to a wrong that she has committed; she is responding to a wrong that she has done *to another*.^9^

In addition, remorse involves the following *evaluative beliefs:*

(iii) ‘Performing X significantly *wronged* the victim, and
(iv) ‘I am blameworthy for wronging the victim through performing act X.’

On this account, remorse is only appropriate in response to offences that can be understood to significantly wrong their victims. For example, whilst it may be appropriate to feel remorse for murder, it is not appropriate to feel remorse for littering on another’s property, even though one might appropriately regret or feel shame for one’s act.

Other writers have suggested that there are further beliefs that accompany prototypical instances of remorse. For example, Raimond Gaita suggests that the remorseful agent (re)acquires a belief about the moral worth of the agent she has harmed as she recognises ‘the reality of [that agent] through the shock of wronging her.’^10^ Furthermore, Michael Proeve and Steven Tudor suggest that the experience of remorse involves the belief that one has ‘become something other.’^11^ However, whilst these sorts of beliefs may accompany many agents’ experiences of remorse, it is somewhat contentious to claim that they must constitute *necessary* features of remorse. Whilst we agree that the victim of the wrongdoing will be of great salience in the offender’s mind, we only posit (i), (ii), (iii), and (iv) as beliefs necessary for the minimal account of remorse that we are invoking here.

Whilst beliefs about one’s role in wronging another and one’s blameworthiness for it are central to the moral value of remorse, they are neither sufficient for morally-valuable remorse, nor exhaustive of the phenomenology of remorse. Indeed, we shall suggest below that, on one plausible interpretation, it might be claimed that an offender cannot be said to truly hold the above evaluative beliefs if these beliefs are not accompanied by the affective dimensions of remorse. We turn now to examine the affective element.

### The Affective Element

In referring to the ‘affective’ elements of remorse, we intend to refer to the feelings that partly constitute an experience of remorse. Although it is difficult to identify necessary, as opposed to indicative, affective elements of remorse, we may say that remorse is a painful emotion, commanding sorrow for the situation, compassion for one’s victim, and self-loathing. Adam Smith famously wrote that, of ‘all the sentiments than can enter a man’s breast, [remorse] is the most dreadful.’^12^ In a similar vein, Proeve and Tudor characterise the affective element of remorse as involving ‘feelings of internal turmoil,’^13^ whilst Jeffrie Murphy suggests that remorse goes beyond powerful guilt insofar as it incorporates intense feelings of hopelessness.^14^ We may say then that the affective element of remorse roughly involves (at least) three objects, with different emotions directed at each. The agent responds emotionally to the *plight of the victim* with sympathy, to *her own malevolence* with self-hatred, and to the *wider situation* with sorrow, despair and perhaps fear.

### The Motivational Element

Finally, remorse produces motivations to carry out particular sorts of acts – motivations that the offender herself endorses. First, the genuinely remorseful offender will hold the intention not to do X again. It should be noted that it is not necessary that the agent is *successful* in refraining from repeating X for her remorse to be genuine. Although repetition of the act would cast the genuineness of the offender’s intentions into doubt, it is possible that an agent may genuinely intend to refrain but is unable to succeed in doing so. For instance, suppose that an offender meets the cognitive and affective conditions of remorse and forms a genuine intention not to re-offend. At a later time though, she feels the pull of a compulsive desire to commit a similar offence; although she would repudiate this desire if she could, it is nonetheless effective in moving her to act. We believe that it is plausible to claim that such an offender may nonetheless feel genuine remorse for her initial crime.

The second motivational element is the desire to atone. The specific nature of ‘atonement’ will depend on the facts of the particular case, but atonement will most often involve the agent trying to apologise and to offer some sort of reparation to the victim. Even where the wrong cannot be repaired, the remorseful offender will desire to ‘do something’ to try to make up for the harm caused. Again, we can see that this desire to atone is contingent on holding the beliefs that there is something to atone for—that the victim suffered harm that one culpably caused. Further, similar caveats apply to the desire to atone as to the offender’s intention not to repeat the offence: although it would cast doubt on the genuineness of the remorse if the offender did not make any attempt to offer some sort of material or symbolic reparation, a rebuffed apology or refusal to accept reparation will not automatically speak against the genuineness of the agent’s remorse.

## Neurointerventions and Remorse

In view of the above taxonomy of the elements of remorse, it seems that neurointerventions with three types of effect are pertinent to our discussion. We shall begin by considering neurointerventions with promnesic effects; such interventions might plausibly have an effect on an agent’s capacity to feel remorse insofar as an offender must have the relevant memories of her wrongful act in order for her to be able to hold the beliefs that constitute the cognitive element of remorse. Second, having suggested that the offender must in many cases have some capacity for empathy in order to be capable of holding the beliefs that constitute the cognitive element of remorse, as well as experiencing the aversive affect that constitutes the affective element, we shall consider neurointerventions that might enhance empathy. Finally, we shall consider interventions that might affect the recipient’s motivations.

Prior to explicating these neurointerventions, it should be acknowledged that there are important practical barriers to using the interventions that we list below in the criminal justice system. Our interest in these interventions is thus not grounded in the belief that they represent currently practicable ways in which to facilitate remorse; rather, we believe that they indicate the potential for future neurointerventions that might have such effects. However, we believe that it is incumbent upon us to consider the philosophical issues that such neurointerventions might raise, even if such technologies are not *currently* available. Furthermore, we believe that considering the facilitation of remorse through the use of such interventions can lead to important insights about the nature and value of remorse itself.

### Memory

An offender’s ability to recall a past set of events in which she was involved, or her ‘autobiographical episodic memory,’ is particularly important for the cognitive element of remorse. One reason that autobiographical memory is important is that it provides grounds for the offender to hold the non-evaluative belief that she performed X. However, it seems that this belief could also be grounded by the testimony of others who witnessed the offender’s performance of X. Nonetheless, autobiographical memory is still likely to be important with respect to an agent’s capacity to feel remorse in such cases, insofar as holding the belief that ‘one is blameworthy for X’ will often require that one has access to one’s intentional states at the time of performing X. Whilst the testimony of others may provide one with sufficient grounds for believing that one performed some act, it may not provide one with sufficient grounds for believing that one was blameworthy for that act.

In the case of Maciej Zientarski that we delineated at the beginning of the paper, the offender’s loss of autobiographical memory was caused by direct physiological damage to his brain; retrograde amnesia caused by such damage is commonly termed ‘organic’ retrograde amnesia.^15^ Following ‘Ribot’s Law,’ organic retrograde amnesia commonly impairs recently formed memories to a greater extent than older memories.^16^ As such, if an offender suffers from organic retrograde amnesia as a result of a traumatic brain injury sustained during her offence, it is likely that this amnesia will involve the loss of the autobiographical memories that are important for remorse; namely, memories pertaining to her carrying out her offence.

Alternatively, some offenders may suffer from *dissociative* amnesia for their acts; in contrast to organic amnesia, dissociative amnesia is understood to have a *psychological* rather than physiological cause. Dissociative amnesia is a highly contested phenomenon. Many theorists are sceptical about whether it is actually possible,^17^ whilst even those who believe that it is possible disagree about the psychological mechanisms that underlie it.^18^ We lack the space to adequately explore these avenues of disagreement here. For the purpose of this paper, we shall follow Andrew Moskowitz’s assessment that, whilst some dissociative amnesia claims are undoubtedly simulated, it is unlikely that the majority are.^19^ It is also worth noting that claims of amnesia for criminal offences are relatively common. A recent review suggests that approximately 25–40 % of those who are found guilty of homicide claim to be amnesic for the offence.^20^

As long as an amnesic offender did not suffer from a failure in initial memory encoding, then it may theoretically be possible to facilitate memory retrieval in various ways.^21^ In fact, the criminal justice system already uses methods intended to facilitate retrieval in criminal offenders and eyewitnesses of crime. Historically, hypnosis has been used extensively as a witness memory enhancement procedure in a number of jurisdictions.^22^ In recent times, the use of hypnosis has been superseded by the ‘cognitive interview technique,’ which incorporates several mnemonic devices that are intended to facilitate retrieval.^23^

Recent technological advances have raised the prospect of using pharmacological means of facilitating retrieval. For instance, it has been claimed that 5-HT6 receptor antagonists can produce promnesic effects in diverse conditions,^24^ whilst Donepezil has been shown to significantly improve long-term visual episodic recall.^25^ A recent study has also suggested that Acetylcholine facilitates recovery of episodic memory after brain damage.^26^

In addition to these pharmacological agents, there has been growing interest in using other neuro-technologies to facilitate memory retrieval. Trans-cranial direct current stimulation has not only been used to modulate long-term episodic memory,^27^ but also to selectively enhance the memories of specifically valenced kinds of stimuli.^28^ As Barbara Penolazzi et al. point out, such stimulus specific protocols could be fundamental to improving “eyewitness memory and the recovery of different kinds of patients (like amnesic or depressed people) from an impaired processing of emotional stimuli.”^29^ Finally, researchers have recently become interested in the possibility of using deep brain stimulation to facilitate memory retrieval,^30^ following a recent study that found that bilateral hypothalamic deep brain stimulation can have profound effects on autobiographical memory.^31^

### Empathy

It is often claimed in lay discussions that remorseless offenders ‘lack empathy.’ Whilst this seems plausible, it is important to be clear about the sense of empathy that one is invoking in this sort of claim. We lack the space to adequately analyse the myriad different approaches to the concept of empathy here.^32^ Rather, we shall assume that empathy involves both cognitive and affective components. Following James Blair, we may say that ‘cognitive empathy’ (or to have a ‘Theory of Mind’) is the ability to represent the internal mental states of others, whilst ‘emotional empathy’ pertains to the extent to which the agent experiences an emotional response to another’s emotional stimulus.^33^

It seems that deficits in either of these components of empathy could have profound effects on the offender’s capacity to feel remorse. For example, in cases of harmful wrongdoing, an offender who has a severe deficit in cognitive empathy may lack remorse because she may fail to comprehend that doing X caused significant harm to another (and thereby wronged that person), insofar as she lacks the ability to have an internal representation of her victim’s experiencing harm.

Alternatively, even if the offender is capable of cognitive empathy, she might nonetheless lack the capacity for remorse because of a severe deficit in emotional empathy. There are two interpretations of how this deficit could undermine the capacity for remorse. First, one might understand this deficit to preclude the offender from experiencing the relevant aversive affect as a result of her belief that her doing X caused harm to another. Second, one might advance the stronger claim that the lack of aversive affect in response to causing such harm evidences the fact that the offender lacks the moral concepts that are necessary for holding the belief that her doing X was morally wrong in the first place. We shall explore this latter interpretation in more detail in Sect. 3.^34^

Whilst extreme cases such as these may be rare, it seems plausible to claim that some criminal offenders have deficiencies in empathy that prevent them from experiencing genuine remorse. For instance, a number of studies have suggested that individuals with autism and Asperger’s syndrome have an impaired theory of mind,^35^ to an extent that compromises their ability to experience remorse^36^; furthermore, recent studies have suggested that the prevalence of sufferers of such disorders in forensic samples is substantially higher than in general community samples.^37^ Moreover, it has been suggested that the lack of empathy that is characteristic of many sex offenders can often be explained by underlying deficits in the offender’s theory of mind.^38^ Conversely, whilst studies suggest that psychopathic criminal offenders do not exhibit theory of mind deficits, Blair has argued that the psychopath’s lack of empathy is best explained by the hypothesis that psychopaths have severe deficits in elements of emotional empathy.^39^

These examples are not intended to suggest that an offender who lacks remorse for her crime due to a lack of empathy for her victim must thereby suffer from some sort of pathology. Rather, it seems that empathy admits of degrees, and that some remorseless offenders may not be completely devoid of empathy, even though their level of empathy for their victims is insufficient to make them experience remorse for their offences.^40^ In such cases, it might be possible to intervene to facilitate an experience of remorse by enhancing elements of the offender’s empathy. Indeed, as Mark Carich et al. point out, the promotion of empathy among sex offenders is “a core component in the majority of programs in Britain and the United States,”^41^ and they argue that ‘victim empathy’ can be learned by offenders who display a minimal capacity for empathy.^42^

It seems plausible to claim that neurointerventions could also be used to facilitate remorse amongst offenders who have empathy deficits. Consider first cognitive empathy. A number of studies have suggested that increased oxytocin levels can improve empathic accuracy of mental state attribution in individuals who are less socially proficient.^43^ Furthermore, in view of fMRI research that has isolated brain regions specific to theory of mind,^44^ there has been a case report of the successful use of transcranial magnetic stimulation to the bilateral medial prefrontal cortex to improve social relating and improved understanding in a subject with high-functioning autism spectrum disorder.^45^

Recent research has also suggested that we may be able to use neurointerventions to enhance emotional empathy. For instance, Molly Crockett et al. have shown that increasing serotonin in prefrontal areas can increase aversion to the prospect of harming others in healthy volunteers.^46^ Furthermore, studies of psychopathy have suggested that abnormalities in the pathways between the pre-frontal cortex and limbic system may be responsible for deficits in affective empathy amongst psychopaths.^47^ These findings suggest that it might be possible to modulate affective empathy by stimulating these areas of the brain.^48^

It should be acknowledged that there are likely to be limitations to the extent to which neurointerventions can be used to enhance emotional empathy amongst those who score highly for psychopathic traits. As Dirk de Ridder et al. point out, “… for a neuro-stimulator to function it requires enough target cells to be present,”^49^ and in cases where the psychopath’s lack of empathy is attributable to a severe atrophy in the orbito-frontal cortex or amygdala, there may simply be too few neural substrates for an intervention to have any beneficial effect.^50^ Whilst this may be true, as we mentioned above, it is far from clear that all remorseless offenders lack empathy to this pathological degree.

### Motivational

Pharmacological agents have already been used to alter criminal offenders’ motivation to re-offend. For instance, chemical castration is used in a number of jurisdictions to prevent recidivism amongst sex offenders, and this has been found to have some effect in small-scale studies.^51^ Moreover, as Thomas Douglas points out, its seems likely that the range of medical interventions that are capable of preventing recidivism is likely to expand as neuro-scientists continue to uncover the neural correlates of dispositions that are associated with criminal offending.^52^

In the model of remorse that we delineated above, we stated that the remorseful offender must hold a genuine intention not to re-offend, but that this does not entail that she must be successful in refraining from re-offending. However, it seems that, for an offender’s remorse to be genuine, she must not only hold an intention not to re-offend, she must also take steps to avoid predictable lapses in this intention. To illustrate, suppose that an offender was aware that a certain sort of stimulus reliably triggered a compulsive desire to commit a certain type of offence. We would doubt that such an offender’s remorse for her previous offence was genuine if she continued to expose herself to this stimulus when doing so was avoidable, even if she held an intention not to re-offend.

With these reflections in mind, it seems that neurointerventions that affect the offender’s motivation to re-offend might be relevant to the *expression* of remorse. Although we lack the space to elaborate the position here, it might be claimed that an offender’s agreeing to undergo a neurointervention that could effectively eliminate her motivation to re-offend would be a reliable indication of the genuine nature of her remorse. For instance, it seems that remorseful sex offenders could go some way to adequately expressing their remorse by agreeing to undergo chemical castration.

## Remorse and Criminal Justice

We now turn our attention to the primary question that this paper addresses: should we mitigate the sentence of a remorseful offender whose remorse has been facilitated by the use of neurointerventions? That is, should technologically-facilitated remorse be honoured such that it is permitted the same penal significance as standard instances of remorse?

To focus our discussion, it is useful to consider briefly the grounds for the general claim that we should mitigate the sentence of a remorseful offender in the standard case. We lack the space here to distinguish and analyse all the possible arguments for remorse-based mitigation^53^; rather, our intention here is to provide a brief overview of ways in which one might justify this practice. This will allow us to identify the justification that is most likely to challenge the hypothetical role for neurointerventions in facilitating remorse. Of course, regardless of whether any of the views we highlight below are convincing, in practice, an offender’s remorse is considered a relevant factor to take into account in mitigation at sentencing in many jurisdictions, as we pointed out in the introduction.^54^ Finally, we shall not address the epistemological challenge faced by advocates of remorse-based mitigation regarding how courts can reliably establish that an offender is experiencing genuine remorse.

On a consequentialist justification of remorse-based mitigation, this practice would be justified where it is likely that the offender’s remorse is indicative of a reduced need for specific deterrence or increased prospects for rehabilitation. Here, though, we shall be interested in a retributive justification of remorse-based mitigation. Of course, there are a number of ways in which one might seek to offer a retributive justification of remorse-based mitigation. On a classical retributive view, it might be claimed that, if remorse is aversive enough to constitute suffering, then it may constitute or substitute for some of the deserved punishment. Alternatively, theorists who claim a legitimate role for mercy in retributive penal theory may argue that remorse justifies leniency grounded on a charitable concern for the wellbeing of the offender.^55^ Communicative theorists—whose theories hold that punishment communicates to the offender the deserved message of censure—can justify remorse-based mitigation on the grounds that penal dialogue should be responsive to the offender’s antecedent endorsement of the message of censure.^56^

For the purposes of our discussion, we shall focus on a broad retributive justification for remorse-based mitigation that is sensitive to the moral value of the offender’s remorse. According to this view, remorse-based mitigation is justified insofar as mitigation serves as a deserved form of response to the *moral value* of the offender’s remorse. Call this the ‘moral value’ view of the justification of remorse-based mitigation. Although we cannot adequately defend the claim here, we believe that this framework offers the most plausible grounds for remorse-based mitigation.^57^

Of course, it could be argued that even if enhancing remorse is in some way problematic on the moral value view, we might nonetheless have other consequentialist reasons to mitigate sentences on the basis of enhanced remorse that outweigh these considerations; suppose, for instance, that the empirical data suggested that remorseful offenders are less likely to re-offend. Consequentialists might argue that this consideration could outweigh the moral value view’s arguments against mitigating sentences on the basis of enhanced remorse, even if the latter arguments were convincing.

Nonetheless, we shall examine the issue through the lens of the moral value view for two reasons. First, even if there are strong consequentialist reasons in favour of mitigating sentences on the basis of enhanced remorse, the claim that ‘enhanced remorse would be morally problematic in a way that natural remorse is not’ is one that many are likely to find *prima facie* appealing, even if we assume that both forms of remorse are equally conducive to the goods that the consequentialist values. Whilst this intuition is in tension with the consequentialist approach, the moral value view potentially provides a theoretical basis for it. As such, we shall frame our discussion through the lens of the latter view, as doing so will better enable us to examine and test our intuitions about whether natural remorse is preferable to enhanced remorse, independently of its consequences. Second, we believe that the moral value view offers the strongest grounds for an objection to honouring remorse that has been facilitated through the use of neurointerventions. We therefore situate our discussion within such a framework, so that we can confront what we take to be the most serious objection to the honouring of such remorse in the criminal justice system.

If the view that remorse is morally valuable is to be plausible, it seems that one must first establish that *any* emotional experience can be morally valuable. Such a view is apparent in Aristotle’s writing. In *The Nicomachean Ethics* he claims that moral virtue is “concerned with passions and actions” and that to feel emotions:

… at the right times, with reference to the right objects, towards the right people, with the right motive, and in the right way, is what is both intermediate and best, and this is characteristic of virtue.^58^

Susan Stark further develops this Aristotelian thought by arguing that emotions are morally valuable and necessary to virtue because “… feeling emotions properly represents an agent’s full and proper response to the moral features that there are.”^59^

On this virtue-based approach to grounding the moral value view, the next step is to establish that remorse can be an appropriate emotional response of the sort that is characteristic of moral virtue. Although some writers have denied this sort of claim,^60^ it seems that remorse is a good candidate for a morally-valuable emotion. Recall that for Stark the value of feeling an appropriate emotion is constituted by the fact that it represents an agent’s full and proper response to ‘the moral features that there are.’ It seems highly plausible to claim that remorse represents such a full and proper response; in remorse, the agent both recognises and appropriately responds to an important moral feature, namely, her act of wronging another.

Setting aside objections to this justification for mitigating an offender’s sentence on the basis of remorse *per se*, with this background in mind we can now consider whether there are grounds for claiming that it would be problematic to mitigate a sentence on the basis of remorse that is facilitated by a neurointervention on this justification. In order to focus our discussion, we shall make the following two assumptions throughout.

First, in order to circumvent concerns about the permissibility of non-consensual medical interventions, we shall assume that the offender has provided valid consent to undergoing the interventions we consider. Furthermore, it might be argued that some of the interventions considered above might be less efficacious in facilitating an experience of remorse in an offender who is subjected to them involuntarily. The question of whether this would be the case is itself an interesting theoretical question. However, answering it would require making empirical speculations about the interventions that we cannot adequately explore here. Moreover, we do wish to obfuscate the question of whether it might ever be morally permissible to use these technologies in the criminal justice system by allowing for the possibility that they might be used on offenders non-consensually in a way that might undermine the intervention’s efficacy. We note that, if this were the case, it would add a further complicating factor to the moral analysis that we undertake here.

Second, we shall assume that the offender’s motivation to consent to undergoing the intervention is that she has a non-instrumental desire to feel remorse for her wrongdoing. We make this assumption since an advocate of the moral value view might argue that if the offender’s desire to feel remorse was grounded only by a desire to have her sentence mitigated (thus rendering her desire to feel remorse an *instrumental* desire), this would be sufficient to undermine the genuineness of the ‘remorse’ that the offender felt following the interventions. Whilst these assumptions may to some extent limit the scope of the potential practical applications of our arguments, as we mentioned in the introduction, we are interested here only in establishing the theoretical plausibility of facilitating morally-valuable remorse through the use of neurointerventions in at least some cases.

This, though, is not to deny the importance of the questions raised by the possibility of inducing remorse in people who have only an instrumental desire to feel it, perhaps because they want to ensure that they receive more lenient sentences, for example. The question of whether the criminal justice system should honour such remorse is one of the central questions that would have to be addressed if the use of the technologies described above became practicable. However, prior to addressing the question of whether the offender’s *motivation* in undergoing such enhancement can undermine the moral value of the remorse to which it leads, we should, it seems, first consider the theoretically prior question of whether an advocate of the moral value view could allow for the possibility of enhanced remorse being morally valuable *in any case*. After all, the reasons that an advocate of this view might cite in rejecting the practice of an individual enhancing her capacity for remorse on the basis of a non-instrumental desire to feel remorse would also apply to the practice of her doing so on the basis of an instrumental desire to feel remorse. Thus, in setting aside considerations pertaining to enhancing an offender’s remorse on the basis of her instrumental desire to feel remorse, we are setting aside the question of whether such a motivation might provide sufficient ground for us to refrain from particular instances of enhancing remorse where the offender is motivated by those particular considerations, in order to consider the broader question of whether there are reasons to refrain from enhancing remorse more generally, even if the individual is undergoing enhancement for the appropriate reasons.

With these assumptions in mind, it seems that the moral value justification of remorse-based mitigation can ground a strong objection to honouring remorse that has been facilitated by a neurointervention, insofar as it may be plausible to claim that neurointerventions cannot give rise to genuine, morally-valuable remorse. To begin explicating this sort of objection, consider first the claim that the genuineness of some moral properties depends on the process by which they were acquired. This seems particularly true of the virtues; it seems plausible to suggest that an agent genuinely exhibits a virtue only if she has acquired virtuous dispositions in an appropriate way, say through education and habituation.^61^ As John Martin Fischer and Mark Ravizza point out, on this view, it would be conceptually incoherent to claim that we could develop a ‘virtue pill’ that could induce dispositions that would amount to virtues. They write, “Whereas these pills might induce the pertinent propensities, these propensities would not count as virtues insofar as they were not acquired in the required fashion.”^62^

It might be argued that a similar thought might apply to the use of neurointerventions to facilitate remorse. To illustrate, consider the possibility of a ‘remorse pill’:

*The Remorse Pill*: Assume that the offender believes that she culpably performed X, and that she believes that this was wrong, but that she does not feel bad about this. She then takes a pill that directly induces a negative emotional affect, and a motivation to refrain from doing X again.

It seems plausible to claim that the offender in this case could not appropriately be described as experiencing genuinely morally-valuable remorse even though, *ex hypothesi*, she meets the criteria for all the elements of remorse that we delineated in the first section of the paper. This example might raise an important objection to the potential role of neurointerventions in facilitating remorse, since one might go further and claim that there is no morally significant difference between the remorse pill and the use of the neurointerventions discussed above.

However, we believe that this latter move is too quick. To see why, it is important to be clear about *why* the remorse pill could not be used to generate genuine morally-valuable remorse; more specifically, we need to consider why the ‘remorse’ of the offender who has taken the remorse pill has not been, in Fischer and Ravizza’s terms, ‘acquired in the required fashion.’

One notable feature of the negative emotional state and associated motivations that the remorse pill brings about is that they are divorced from what we have described as the cognitive element of remorse. Although the offender in our example of the remorse pill held the belief that doing X wronged another, this belief played no role in the generation of the offender’s negative emotional state and associated motivations; rather, these were simply pharmacologically induced by the pill in a manner that was unrelated to the agent’s holding these beliefs. It seems plausible to claim that this is why we believe that the offender’s remorse is not ‘acquired in the required fashion.’ In fact, to return to an issue that we highlighted in Sect. 2, it might be suggested that the absence of the aversive affect prior to taking the remorse pill brings into question whether the offender truly holds the evaluative beliefs that are also necessary for remorse. To see why, consider the following claims made by Martha Nussbaum:

The agent who discerns intellectually that a friend is in need or that a loved one has died, but who fails to respond to these facts with appropriate sympathy or grief clearly lacks a part of Aristotelian virtue. It seems right in addition to say that a part of discernment or perception is lacking. This person doesn’t really, or doesn’t fully, see what has happened, doesn’t recognise in a full-blooded way or take it in … The emotions are themselves modes of vision or recognition. Their responses are part of what … truly recognising or acknowledging consists in.^63^

We might compare Nussbaum’s claims here with Alison Hills’ analysis of moral understanding. Hills claims that it is possible to have moral knowledge without having moral understanding, insofar as it is possible to know that something is morally right or wrong without appreciating the explanation for why this is the case.^64^ A critic of using neurointerventions to facilitate remorse might supplement Hills’ account here with the Aristotelian claim that moral understanding involves not only an appreciation of certain kinds of reasons, but also the experience of appropriate emotional responses to these reasons, and to morally relevant facts. The absence of such an emotional response bespeaks an absence of moral understanding, and this, it might be argued, is not only a central feature of remorse, but also grounds its moral value.

If this is right, then the moral value of remorse might be understood to derive in large part from the relationship between the aversive affect and the beliefs that generate it in a genuine experience of remorse. The beliefs that constitute the cognitive element of remorse provide the object of the aversive affect that remorse involves; remorse involves experiencing an aversive affect *as a response to* one’s belief that one wronged another. The wrong that one committed is the central object of remorse; the aversive affect does not, so to speak, float free from one’s belief about one’s culpability for that wrong. In turn, the experience of this affect focuses the remorseful agent on the underlying beliefs themselves, underscoring the agent’s moral understanding of her situation. Such interplay of the cognitive and affective elements in a genuine experience of remorse evidences a deep moral understanding of one’s wrongdoing;^65^ in experiencing the pain of remorse, the offender deeply understands what *she* has done. On this view, it is not merely the affective element of remorse to which we attribute value, it is remorse conceived as a response that also involves moral understanding, which the affective element helps to elucidate.

The remorse pill example shows the problem with attempting to capture the essence of remorse by simply identifying and distinguishing its cognitive, affective and motivational elements. In order to adequately capture the value of the moral understanding that remorse involves, we must also attend to the interrelations between these different elements. To put it simply, it is insufficient for genuine, morally-valuable remorse that the agent believes that she performed a certain act, ‘feels bad,’ and is motivated not to act in certain ways; one’s feelings (and motivations) must be grounded by one’s belief that one has acted in a way that has caused harm to another, and that this was morally wrong.

Accordingly, it seems that there might be plausible grounds for claiming that we should not honour the remorse that is experienced by an offender who took the remorse pill on the moral value view of remorse-based mitigation. If these conclusions regarding the incompatibility of the remorse pill and genuine remorse should carry over to the use of the neurointerventions to facilitate remorse, then it must be the case that such neurointerventions would similarly undermine the morally-valuable understanding that remorse involves, by similarly divorcing the affective and motivational elements of remorse from the cognitive element.

Yet it is not clear that this would be the case. This is most obvious when the neurointervention in question simply enables the agent to hold the non-evaluative beliefs that are relevant to remorse. Consider the use of promnesic interventions that might allow an amnesic offender to retrieve memories of her wrongdoing. Even if an offender came to hold the relevant non-evaluative beliefs about her offence on the basis of such an intervention, it is unclear why those beliefs would not be able to play the same role in generating the affective elements of remorse that they would have played had they been formed naturally in the absence of this sort of intervention. An offender who undergoes a promnesic intervention would only feel remorse if she also comes to hold the relevant evaluative beliefs and forms the appropriate affective response to these newly acquired beliefs. As such, we might say that a promnesic intervention enables an offender to hold the non-evaluative beliefs without having a direct influence on whether those beliefs will generate the relevant evaluative beliefs and appropriate affective response.

If this is right, then a promnesic intervention may facilitate the offender’s experience of remorse in a manner that is compatible with its moral value. In contrast, we can understand the remorse pill in the above example to go beyond the mere facilitation of remorse, since it directly *induces* the affective element of remorse in a manner that disconnects the affective and cognitive elements of remorse. The problem with inducing remorse in this way is that, in disconnecting these elements of remorse, we undermine its moral value.

It might be objected that an intervention that affects the offender’s empathy would amount to an intervention that induces rather than facilitates remorse on this approach. Again, though, this is too quick; empathy enhancements need not necessarily rupture the connection between the cognitive and affective elements of remorse that undergirds remorse’s moral value. This is most clearly so in the case of interventions that would affect cognitive empathy; such enhancements would have effects on the offender’s ability to experience remorse that are similar in kind to promnesic interventions, insofar as both types of intervention enable the offender to hold a non-evaluative belief necessary to remorse, without implying that that belief will lead the offender to form an appropriate affective response to it, nor, for that matter, the relevant evaluative belief. One can fail to hold the belief that one performed an act that wronged another because one cannot remember doing so; but one can also fail to hold that belief because one cannot adequately conceive of one’s actions affecting the mental life of another.

Emotional empathy enhancements might be understood to be more problematic in this regard. Indeed, if one were to understand the enhancement of emotional empathy as simply amounting to imposing an experience of the negative aversive affect that remorse connotes, then enhancements of emotional empathy would be indistinguishable from the remorse pill. However, our above discussion of moral understanding and the moral value of remorse suggest a more complex picture of the role of emotional empathy in remorse. On this picture, even if emotional empathy undergirds an agent’s ability to experience a negative aversive affect in response to another’s emotional stimuli, increasing one’s empathy does not entail that this affect will be experienced. For an individual with a sufficient degree of emotional empathy to experience this affect, she must be presented with an emotional stimulus; in the case of remorse, this stimulus is the belief that she has significantly wronged another. The affect generated by the remorse pill, in contrast, requires no such belief. Furthermore, our above discussion suggests that emotional empathy can also be understood to serve as a mode of moral perception that allows individuals to hold evaluative beliefs that incorporate moral concepts. As we shall now argue, on this interpretation of the role of emotional empathy in remorse, emotional empathy enhancements need not disrupt the crucial connection between the cognitive and affective elements of remorse.

As we pointed out in Sect. 2, criminal justice authorities already use empathy training to enhance empathy amongst certain criminal offenders. Presumably, part of the reason that we do not believe that this training undermines the genuineness of the remorse that it might precipitate is that such training does not simply induce the aversive affect that is associated with remorse in a manner that is unrelated to the cognitive element; rather, the hope is that such training may lead offenders to develop emotional responses to their beliefs about their causing others harm in the way that constitutes a virtuous form of moral understanding of the sort we sketched above.

It is not clear why neurointerventions that enhance emotional empathy cannot be understood in a similar fashion. Empathy enhancements need not simply induce an aversive affect in the recipient like the remorse pill; emotional empathy should not simply be equated with the affective experience to which it might give rise. Rather, on the view that we have developed in this section, such enhancements might be understood as providing offenders with the tools to develop an appropriate affective response. Further, as our above discussion of Nussbaum’s view suggests, we may understand the offender who fails to respond emotionally to certain facts to lack a form of moral perception. On this view, enhancing emotional empathy can be understood to involve making this mode of moral perception available to the offender, but it need not determine a particular, all things considered response to what he perceives. By way of analogy, although corrective glasses increase the wearer’s visual clarity, they do not force him to look in a particular direction, nor to find the scene on which his gaze falls attractive. As such, even if the enhanced offender only feels an aversive affect following the neurointervention, this does not entail that deliberative processes have not mediated the aversive affect in any way. Rather, the empathetic offender will only experience the aversive affect insofar as she perceives it to be an appropriate response to her beliefs. The fact that the mode of moral perception underlying this belief is facilitated by a neurointervention does not threaten the crucial connection between the cognitive and affective elements of remorse.

So far, we have suggested that critics might object to honouring remorse that has been facilitated by neurointerventions on the basis that such interventions could not bring about genuine, morally-valuable remorse. Whilst we have argued that such an objection does not apply to the sorts of interventions that we considered in Sect. 2, critics might claim that we have misconstrued their objection. They might suggest that the problem with such interventions is that the remorse they facilitate would not be morally valuable because it is not *authentic* to the agent. We shall conclude by considering the strength of this sort of objection.

Authenticity-based objections are often raised in the neuro-ethics literature. Notably, for our purposes, Nicole Vincent has recently argued against the use of non-consensual neurointerventions for the purpose of restoring an offender’s competence to stand trial, on the basis that they might have profound effects on authenticity and personal identity.^66^ In her discussion, Vincent does not distinguish the effects that neurointerventions might have on the recipient’s personal identity from the effects that they might have on her authenticity. However, the two effects may not be coextensive, and it will be helpful to begin our discussion by explaining why not.

According to prominent psychological accounts of personal identity (understood in the narrative, rather than numerical sense),^67^ the continuity of certain psychological connections (such as the agent’s memories, values and character) is central to personal identity.^68^ With this in mind, an agent can retain her identity following a neurointervention if she retains a sufficient number of psychological connections. However, this intervention may nonetheless generate an effect that is inauthentic to that agent, if the effect is not true to her ‘real’ self, or the stable values, beliefs and dispositions that may be understood to partly constitute her character.

In contrast, if an intervention served to sever a sufficient number of the recipient’s psychological connections, then we might plausibly say that the agent has been so radically changed that she is no longer the ‘same person’; however, the effects of the intervention may nonetheless be authentic to the agent who *now* exists. Such radical neurointerventions could be understood to serve as a perverse form of capital punishment, insofar as they terminate the existence of the agent (in a narrative sense) who committed the offence. However, as we shall suggest below, insofar as memories constitute a psychological connection that can ground personal identity, promnesic interventions might be understood as strengthening identity on a psychological account, rather than undermining it.

The application of an objection based on concerns related to authenticity and personal identity is particularly interesting in this context because, even in standard cases of remorse, the remorseful offender is understood to have undergone a significant change that can be understood as an abandonment of important aspects of her previous self; experiencing remorse inherently involves a rejection of past conduct, intentions and attitudes. Yet we presumably believe that remorse can be authentic to the agent in the normal case; moreover, as we pointed out in Sect. 1, experiencing remorse for the acts of someone else is incoherent—accordingly, if remorse is to be conceptually possible, it must be possible for a person to retain her identity, despite having undergone the drastic change that remorse entails. Accordingly, the challenge for the objection in this context is to explain why the drastic changes that remorse involves are not themselves a threat to the agent’s identity or authenticity, whilst also explaining why the use of neurointerventions to facilitate such changes is problematic in either of these regards.

In order to get clearer on this, it is prudent to say a little more about the concept of authenticity. Broadly speaking, there are two ways in which one can understand authenticity. First, consider what may be described as an ‘essentialist’ understanding of authenticity, according to which authenticity is a matter of *self*-*discovery*. To live authentically on this account is to live in accordance with one’s essence, or to be true to an extant and mainly static self. In contrast, an ‘existentialist’ understanding of authenticity suggests that authenticity can be a matter of *self*-*creation*; to be authentic on this account is to consciously shape one’s own characteristics within the constraints imposed by the self’s enmeshment with social and genetic factors.^69^

The existentialist approach to authenticity is broadly amenable to the use of enhancement technologies to change aspects of oneself; indeed, on the existentialist understanding of authenticity, the offender who voluntarily chooses to undergo an neurointervention in order to facilitate an experience of remorse can be understood to be taking steps to engage in the self-creation that is central to authenticity.^70^ As such, the authenticity-based concern with using (consensual) neurointerventions to facilitate remorse may not arise if one endorses the existentialist understanding of authenticity.

Furthermore, it might even be argued that the essentialist understanding cannot ground a strong objection to this practice. As Neil Levy has argued, the essentialist view of authenticity is compatible with an agent’s use of enhancement technologies to change aspects of herself, insofar as engaging in a project of self-discovery can lead one to the realization that one may need to change oneself in order to become ‘who one really is’ on the essentialist understanding.^71^ On the assumption that a non-remorseful offender harbours a non-instrumental desire to experience remorse for her criminal act for its own sake, and consents to undergoing a neurointervention on that basis, it seems that she might plausibly be understood as taking action to align their outer self with ‘who she really is’ in the manner that Levy argues is compatible with the essentialist understanding of authenticity.^72^

Nonetheless, we suspect that critics will claim that there is something more to the essentialist’s worry in the present context. To illustrate, consider again the example of the remorse pill. Even if, contrary to our analysis above, the remorse pill could be understood to generate an experience of morally-valuable remorse, it seems that some opponents might nonetheless claim that the remorse pill would still be problematic for the recipient’s authenticity. The pill would, it might be claimed, threaten authenticity by creating a disjunction between the person who committed the offence, and the person who feels the remorse.

Whilst this objection has a different focus than the objection pertaining to the ‘genuineness’ of the remorse that neurointerventions might facilitate, we believe that similar considerations can be raised against it. As we suggested above, the problem with this objection at first glance is that even in standard cases of remorse this sort of disjunction can be understood to obtain in some sense. Accordingly, to increase the plausibility of the objection, it seems that its proponents need to claim that the remorse pill differs from the standard case in some way. For instance, it might be claimed that the change that the remorse pill brings about could not be seen as a result of a process of ‘intelligible’ personal development.^73^

A general problem with authenticity-based objections to enhancement technologies that appeal to the notion of ‘intelligibility’ is that the latter notion is a somewhat nebulous concept^74^; that is, it is unclear precisely what it is that makes a change ‘intelligible’ to an agent in the manner that makes the change acceptable from the point of view of authenticity. Yet, even in the absence of a detailed account of intelligibility, in view of our discussion of moral understanding above, it seems that the interventions that we are considering in this paper would not render the remorse that they facilitate unintelligible on a plausible account of intelligibility.

Consider first promnesic interventions. Enhancing an agent’s ability to access lost memories would serve to reconnect her with her earlier self at the time the memories were encoded. If restoring such memories leads to the agent being able to feel remorse, this results from the *restoration* of psychological connections; the remorse here would be intelligible to the agent insofar as it is generated by memories that she can understand as ‘hers.’ This is a striking implication in view of Vincent’s authenticity-based objection to the use of non-consensual neurointerventions to restore the mental capacities that undergird competence to stand trial. In contrast to these interventions, it seems that the promnesic interventions that we have considered here would not only provide a basis for an intelligible personal development, they would also, if anything, restore key psychological continuities that ground personal identity, rather than undermine them, *even if they were used non*-*consensually*.

Furthermore, as noted above, an intervention that enhances empathy would not necessarily induce particular evaluative beliefs or feelings in the agent; rather, it would provide the agent with the perceptual apparatus required to respond in a compassionate way, without making it certain that she will. The agent must still evaluate the moral properties of her conduct and its consequences. Enhanced empathy plays a role in making these properties more vivid and accessible, but it does not force a new set of values on the agent such that she is rendered disconnected from her earlier self. Indeed, we can imagine the agent, who, having had her empathy enhanced, experiences herself as having her attention directed to facts that were previously opaque to her, in a way that is consistent with intelligible personal development of the sort that critics of enhancement often celebrate, and the sort of moral understanding that we described above.

A further objection could be raised about the potentially transient nature of the effects of neurointerventions. If the effects are only contemporaneous with the operation of the intervention, then the remorse it facilities might be fleeting or reversible in a way that arguably might preclude it from bearing moral value; its disappearance on ‘turning off’ the technology reveals that the remorse facilitated was a mere artifact of the technology, and thus insufficient to command moral or penal value.

We agree that, if it were the case that the agent only felt remorse at the time of the operation of the neurointervention, and returned to her prior, unremorseful state on cessation, we would question the moral value of the remorse. In response to this observation though, we should first acknowledge that the effects of some plausible neurointerventions could be permanent, such that operation of the intervention would not need to continue in order for its effects to persist. This could be the case with the restoration of memory, for example. Other mechanisms, for example, the enhancement of empathy, could indeed be more transient, in the sense that the primary effects only obtain when the intervention is in operation. Here we must ask what remains once the primary effects cease, and determine whether any lasting effects are sufficient to ground the moral and penal value of the technologically-facilitated remorse. In the case of enhanced empathy, although the affective dimension might soften in the absence of the neurointervention, it is plausible that the moral understanding that it enabled could remain. Return to the analogy with corrective glasses. Even when the glasses are removed, the wearer can remember the things she saw and her reactions to them, even if she cannot still see them clearly without the glasses. Analogously, an agent might need enhanced empathy to hold and understand the implications of the belief that ‘performing X significantly wronged the victim,’ but this belief could persist even in the absence of continued acute affect. Affect might be necessary for coming to possess moral understanding but not necessary for its retention.

Indeed, it is instructive to compare this possibility with the evolution and attenuation of natural remorse over time: natural remorse may come and go, and gradually abate in affective strength. Such evolution, however, would not necessarily lead us to question the moral value of the original experience of remorse, nor the value of its more attenuated form. The original remorse has a sustained effect on how the agent thinks about her future actions in the shadow of her past wrong. This continued influence is demonstrative of continued moral understanding.

## Conclusion

We have argued that there are some cases in which different neurointerventions could be used to facilitate genuine, morally-valuable remorse. In doing so, we have offered a partial defence of the claim that, on the moral value justification of remorse-based mitigation, the criminal justice system should, hypothetically, honour remorse that has been facilitated by neurointerventions in the same way that it honours naturally-occurring remorse.

Of course, our arguments here have somewhat limited scope. We have lacked the space to defend the moral value justification of remorse-based mitigation, and we have not analysed objections that might be raised against facilitating remorse through the use of neurointerventions by alternative accounts of this justification. Moreover, our conclusions in this regard rely on assumption that the offender in question has consented to undergoing the neurointervention on the basis of a non-instrumental desire to experience remorse. It is unlikely that this assumption would reflect all real-world cases in which neurointerventions might 1 day be used. The question of whether the criminal justice system should honour an experience of remorse that an offender has intentionally caused herself to have for only instrumental reasons is a central practical question that our discussion here raises, and is one that would need to be addressed before implementing these technologies in a real-world setting.

Despite these limitations, we believe that we have defended the practice of using neurointerventions to facilitate remorse from objections that many would take to be powerful criticisms. In addition to addressing these objections, our discussion has served to illuminate more precisely the way in which advocates of the moral value view of the justification of remorse-based mitigation should understand the nature of remorse’s moral value. Through considering how remorse can plausibly be facilitated by neurointerventions, we are able to see more clearly what is required for a morally-valuable experience of remorse, and how this value is affected by the way in which the remorseful experience is brought about. Accordingly, our conclusions are significant for those interested in the role of remorse in sentencing more generally.

## Footnotes

1 See http://www.fakt.pl/Skazany-dziennikarz-motoryzacyjny-Maciej-Z-u-Hanny-Lis-w-Pytaniu-na-sniadanie-w-TVP,artykuly,194558,1.html.

2 Sentencing Council (2012).

3 Tombs and Jagger (2006), Jacobson and Hough (2007).

4 Sundby (1998).

5 See Douglas et al. (2013), 395–396 for a useful overview.

6 Sell vs United States (2003), Singleton vs Norris (2003). See Vincent (2014), 25.

7 See Nussbaum (2001), Neu (2000).

8 This account echoes the account of remorse that one of us has developed in greater detail elsewhere. See Maslen (2015).

9 Proeve and Tudor (2010), 41.

10 Gaita (2002), 52.

11 Proeve and Tudor (2010), 42.

12 Smith (2002), 99.

13 Proeve and Tudor (2010), 43.

14 Murphy (2014), 140.

15 Stracciari et al. (1994).

16 Ribot (1909).

17 For instance, see McNally (2004), Ornish (2001).

18 See Porter et al. (2001), Payne et al. (2004), Kikuchi et al. (2010).

19 Moskowitz (2004), 35.

20 Christianson et al. (2007), 15. See also Schacter (1986), Taylor and Kopelman (1984), Evans et al. (2009).

21 Kihlstrom and Schater (1995), 341.

22 Wagstaff (2008), 1285. Kleinhauz et al. (1977).

23 Geiselman et al. (1986), Fisher and Edward (1992).

24 Meneses et al. (2011).

25 Grön et al. (2005).

26 Croxson et al. (2012).

27 Manenti et al. (2013).

28 Penolazzi et al. (2010).

29 Ibid.

30 Hu et al. (2009).

31 Hamani et al. (2008).

32 See Cuff et al. (2014) for a review.

33 Blair (2005), 699.

34 For discussion, see Levy (2014), Glannon (1997), Levy (2007), Vargas and Nichols (2007).

35 Baron-Cohen et al. (1985), Baron-Cohen (2000), Hill and Frith (2003).

36 Haskins and Silva (2006).

37 Ibid.; Scragg and Shah (1994).

38 Ward (1997), Keenan and Ward (2000).

39 Blair (2005), Ali and Chamorro-Premuzic (2010).

40 See Proeve and Tudor (2010), 143. See Carich et al. (2003), 258. Moreover, psychopathy can also be understood as admitting of degrees. See Marcus, John, and Edens (2004), Murrie et al. (2007).

41 Carich et al. (2003), 256.

42 Carich et al. (2003), Pithers (1999). Notice also that those suffering from pathological deficits in cognitive empathy often receive training to improve their theory of mind. Hess et al. (2008).

43 Domes et al. (2007), Guastella et al. (2010), Bartz et al. (2010).

44 See Gallagher and Frith (2003).

45 Enticott et al. (2011).

46 Crockett et al. (2010).

47 Blair (2003), Glenn et al. (2009).

48 Hétu et al. (2012), 100, Avenanti et al. (2005), Boggio et al. (2009).

49 de Ridder et al. (2009), 173.

50 See Maibom (2014).

51 Lösel and Schmucker (2005), 128.

52 Douglas (2014), 102.

53 For a detailed analysis, see Maslen (2015).

54 Importantly, the absence of remorse is ordinarily not punished *per se*.

55 See Tasioulas (2006). For criticisms of these views see Tasioulas (2006). For criticisms of these views, see Maslen (2015).

56 See Maslen (2015).

57 See Proeve and Tudor (2010) and Maslen (2015).

58 Aristotle (2007), 41.

59 Stark (2004a), 47. See also Stark (2004b).

60 Bittner (1992).

61 Roberts (1984), 235.

62 Fischer and Ravizza (1998), 182.

63 Nussbaum (1992), 79.

64 Hills (2009).

65 See Wiggins (1991), 196 for a similar view.

66 Vincent (2014), 30.

67 See Schechtman (1996).

68 Parfit (1984).

69 Degrazia (2000), 137.

70 Ibid., 136.

71 Levy (2011), 316.

72 Ibid., 317.

73 Kass (2003), 22.

74 Juth (2011), 43.
